# Associations Between Sex Hormone Levels and Autistic Traits in Infertile Patients With Polycystic Ovary Syndrome and Their Offspring

**DOI:** 10.3389/fendo.2021.789395

**Published:** 2022-01-31

**Authors:** Lijie Jiang, Li Tian, Jiajia Yuan, Xinjie Xu, Fan Qu, Rong Zhang, Jianliu Wang

**Affiliations:** ^1^ Reproductive Medicine Center, Department of Obstetrics and Gynecology, Peking University People’s Hospital, Beijing, China; ^2^ Neuroscience Research Institute, Department of Neurobiology, School of Basic Medical Sciences, Peking University, Key Laboratory for Neuroscience, Ministry of Education/National Health Commission, Autism Research Center of Peking University Health Science Center, Beijing, China; ^3^ Medical Science Research Center, Research Center for Translational Medicine, Department of Scientific Research, Peking Union Medical College Hospital, Beijing, China; ^4^ Women’s Hospital, School of Medicine, Zhejiang University, Hangzhou, China; ^5^ Department of Integration of Chinese and Western Medicine, School of Basic Medical Sciences, Peking University, Beijing, China

**Keywords:** polycystic ovary syndrome, autism spectrum disorder, sex hormone, offspring, autistic traits

## Abstract

**Objectives:**

1) To explore the associations between autistic traits and sex hormone changes in patients with polycystic ovary syndrome (PCOS); 2) To explore the influence of PCOS parental autistic traits and related sex hormone changes on autistic traits in their offspring.

**Method:**

This is a case–control study that recruited two groups: polycystic ovarian syndrome-induced infertile subjects as the observation group and fallopian tube factor-induced infertility subjects as the control group. Both cohorts were patients undergoing infertility treatment in the Productive Medicine Center, Peking University People’s Hospital. Two types of questionnaires were completed by patients between May 1^st^, 2015, and May 1^st^, 2016: 1. The autism-spectrum quotient (AQ) 2. Repetitive behavior scale-revised (RBS-r). Levels of sex hormones in serum were measured in patients. The correlations between the levels of these biochemical factors and scores of the autistic traits were analysed. From July 1^st^, 2020 to September 1^st^, 2021, these patients were followed up by telephone and asked to fill out a questionnaire online. The questionnaire included date of delivery, complications, medicine used and negative events during pregnancy (e.g., death of relatives, divorce, etc.), delivery condition, breastfeeding, AQ scale and Autism Behavior Checklist (ABC) of their children.

**Results:**

The patients in the PCOS group had significantly higher AQ scores than those in the control group. Levels of luteinizing hormone and testosterone were also higher in the PCOS group. No significant differences were found between the two groups in RBS-r levels, follicle-stimulating hormone, estradiol or progesterone. In the two combined groups, there were significantly positive correlations between the AQ scores and the luteinizing hormone concentration, as well as between scores of RBS-r and testosterone concentration. Moreover, there was a significantly negative correlation between the level of progesterone and the RBS-r score. According to the follow-up data, the AQ scores of offspring were positively correlated with the RBS-r scores of their mothers. The ABC scores of offspring were positively correlated with the RBS-r scores and the childbearing age of their mothers. No significant difference was found between the two groups in age of delivery, complications, special medication used, negative events during pregnancy, delivery situation, postpartum breastfeeding, age of children, or AQ scores or ABC scores of children. There were no significant correlations between the scale scores of children and the related sex hormone levels of mothers. This could indicate that the higher levels of luteinizing hormone and testosterone and the lower level of progesterone accompanied more pronounced autistic traits in PCOS. Furthermore, the higher delivery age and RBS-r score in mothers accompanied the higher AQ and ABC scores in children.

**Conclusion:**

Compared with the control group, PCOS patients had more autistic traits (especially social dysfunction). The autistic traits in PCOS patients might be related to the elevation in testosterone concentration and luteinizing hormone levels and the decline in progesterone level. Moreover, the autistic traits in the offspring of PCOS patients might be related to the parental high delivery age and high tendency to autism traits.

## 1 Introduction

Polycystic ovary syndrome (PCOS) is one of the most common infertility causes in women and is mainly characterized by oligoanovulation, ovarian polycystic morphology and hyperandrogenism (1). Some recent studies have demonstrated an increased risk of autism spectrum disorder (ASD) in the offspring of PCOS patients (2–5).

ASD is a neurodevelopmental disease with core symptoms, including social communication disorder, narrow interests, and repetitive behaviors (6). There are many factors in the etiology of ASD, such as genetics (7, 8), environment (9, 10), and some factors in research progress, including metabolism (11), gut microbiome (12), and endocrine (13, 14). Numerous researchers have reported that prenatal exposure to high levels of androgen may increase the risk of ASD in offspring (13–16). It seems that sex steroids might be the potential link between PCOS and ASD. However, previous studies have not explored the autistic traits of PCOS itself and the association with sex steroids. This study aimed to test the relationship between the PCOS family (themselves and their offspring) and autistic traits and to analyse the correlation between sex steroid and autistic trait scores in patients with PCOS.

## 2 Materials and Methods

### 2.1 Subjects

All subjects were recruited between May 1^st^, 2015, and May 1^st^, 2016, from the Reproductive Medicine Center, Peking University People’s Hospital, Beijing, China, and sorted into two groups: an observation group of subjects with infertility due to PCOS [based on Rotterdam diagnostic criteria (1)] and a control group of subjects with infertility due to fallopian tubes. The inclusion criteria were as follows: (1) age between 20 and 40 years old; (2) definite diagnosis issued by the hospital; (3) normal intelligence level and reading ability; and (4) voluntary participation in this study and signing of informed consent. Exclusion criteria included (1) serious physical or mental diseases; (2) pregnancy, lactation, or menopause; and (3) recent (within a month) use of contraceptives or other drugs that affect sex hormone levels. One hundred patients in each group were recruited to complete the AQ (Autism Spectrum Quotient) and the Repetitive Behavior Scale-revised (RBS-r). The sex hormone levels of both groups were measured with peripheral blood samples. Moreover, PCOS patients were also evaluated for acne and hirsutism.

Approximately 5 years later, from July 1^st^, 2020 to September 1^st^, 2021, these 200 patients were followed up by telephone. If their babies were above 2 years old, they were asked to fill out a questionnaire online. The questionnaire included date of delivery, complications, drugs used and negative events during pregnancy (e.g., death of relatives, divorce, etc.), delivery condition, breast-feeding, AQ scale and ABC (Autism Behavior Scale) scale about their children. This study was approved by the Peking University Institutional Review Board (Approval No. IRB00001052-15031).

### 2.2 Instruments

2.2.1 The AQ, designed by Simon Baron-Cohen in 2001, is a self-administered quotient measure that measures autistic personality traits in adults. The score is based on five factors, namely, social ability, attention, communication ability, detail, and imagination. A higher score represents more pronounced autistic personality characteristics (17). RBS-r, designed by Bodfish JW in 1999 (18), mainly targets stereotyped behaviors. ABC, developed by Krug in 1978 (19), lists 57 behavioral characteristics of autistic children, including five aspects: sensory, relating, body and object use, language, and social and self-help. It can be used as an autism screening scale for children aged between 2 and 14 years old. A higher score represents more pronounced autistic traits.

2.2.2 Sex steroids, including luteinizing hormone (LH); follicle-stimulating hormone (FSH); estradiol (E2), progesterone (P); and testosterone (T), were measured in the clinical laboratory of Peking University People’s Hospital.

2.2.3 The acne score was determined by the clinical overall scoring method (20). Six parts of the face were observed: frontal, left cheek, nose, and chin, coupled with the chest and back. The lesion severity was divided into 5 grades: 0, no lesion; 1, ≥1 acne; 2, ≥1 papule; 3, ≥1 pustular; and 4, ≥1 nodular cyst. Multiplying the score of the most serious skin lesion type in each zone results in a total score, with 1-18 as mild; 19-30 as moderate; 31-38 as severe; and > 39 as extremely severe.

2.2.4 In this study, an improved F-G scoring method was used to evaluate hirsutism. According to the amount and distribution of the hair, 0 ~ 4 points were recorded, and a total score ≥ 8 points was used to diagnose hirsutism (21, 22).

### 2.3 Statistical Analyses

Data were managed and analysed using SPSS 25.0. An independent t test was used to compare continuous variables. If the sex hormones data exceeded three standard deviations, we would delete them as the extreme value when we analysed the difference between the two groups. The chi-square test or Fisher’s exact test was used to compare categorical variables. Given the large sample size of the combined two groups (n=53~195), the Pearson correlation coefficient was used to examine the correlation between sex hormone levels in plasma and behavioral scores. All statistical analyses were two-sided, and P<0.05 was considered statistically significant (unless otherwise described).

This study is an observational study, with high uncertainty in estimating the sample size. According to relevant literature reports (23), there were 100 cases in each group of questionnaires, and the sample size of each group of blood samples should reach 30 cases.

## 3 Results

**Figure 1:**
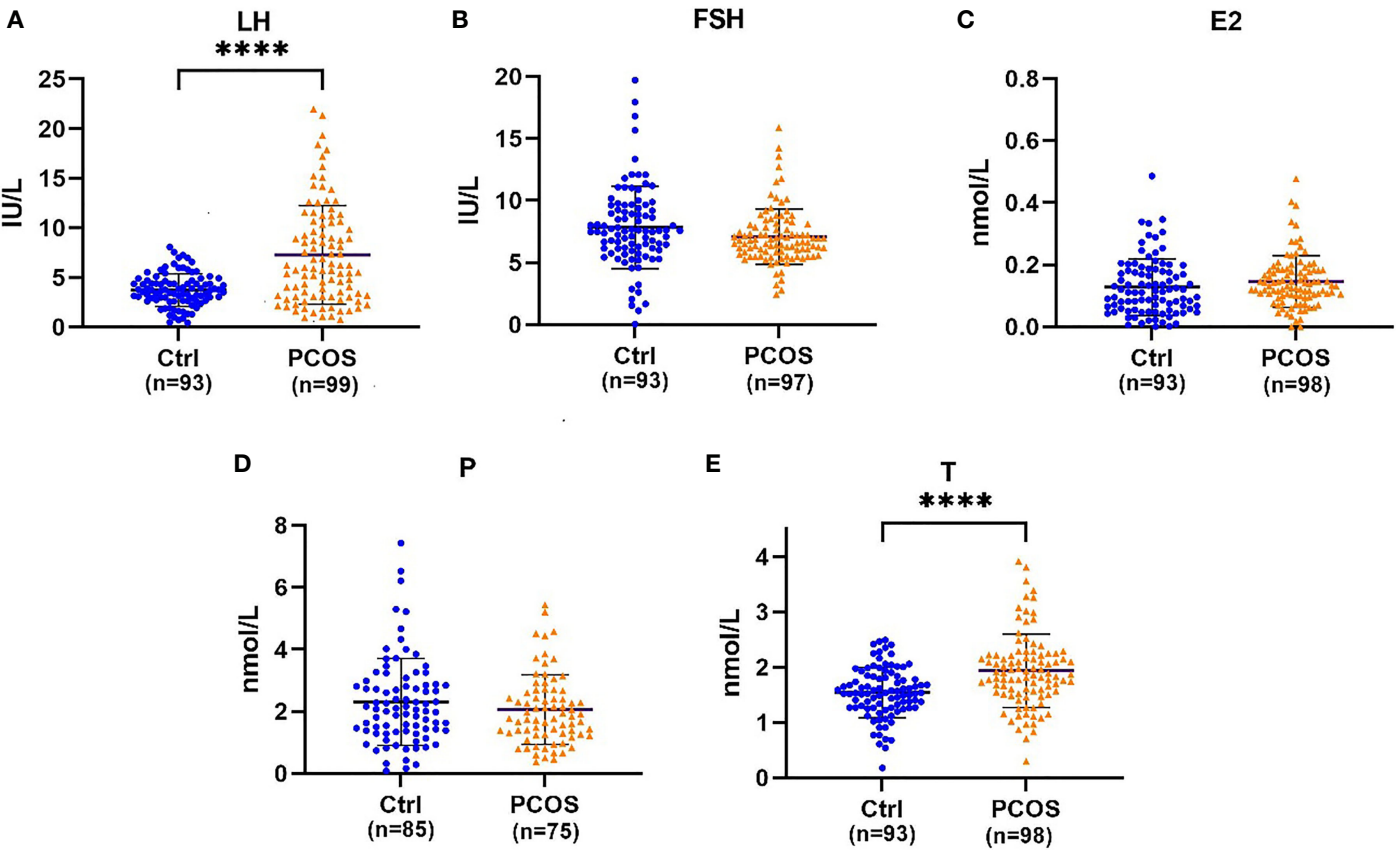
**(A)** Beeswarm plots illustrating the distribution of LH (luteinizing hormone), **(B)** FSH (follicle-stimulating hormone), **(C)** E2 (estradiol), **(D)** P (progesterone), and **(E)** T (testosterone) serum concentrations. Error bars represent the standard deviation, and the black line between two dark lines represents the mean. ****Statistical significance (P < 0.001).

There were no significant differences in RBS scores between groups. However, the PCOS group had a higher body mass index (BMI) and AQ score than the control group (**Table 1**). Furthermore, the PCOS group had significantly higher abnormal scores in social ability, attention and communication ability but not in details and imagination factors in AQ (**Table 1**). The age of the control group was higher than that of the PCOS group. We further analysed the influence of age on the AQ scores (positive data) of the two groups by using covariance and found that age had no significant influence on the AQ scores (P=0.330), but the difference in the AQ scores between the groups was still obvious (P=0.000). Women with PCOS had a significantly higher levels of LH (7.27 ± 4.96 vs 3.73 ± 1.64, P=0.000, **Figure 1A**) and testosterone (1.94 ± 0.66 vs 1.54 ± 0.45, P=0.000, **Figure 1E**) than women without PCOS. No difference was found in FSH (PCOS vs control: 7.08 ± 2.24 vs 7.83 ± 3.31, P=0.069, **Figure 1B**), estradiol (PCOS vs control: 0.15 ± 0.08 vs 0.13 ± 0.09, P=0.150, **Figure 1C**), or progesterone (PCOS vs control: 2.06 ± 1.12 vs 2.30± 1.40, P=0.226, **Figure 1D**). Further correlation analysis revealed that there was a significant positive correlation between the total AQ score and LH concentration as well as between the RBS-r score and total testosterone and a significant negative correlation between the RBS-r score and progesterone. However, there were no significant correlations among the total AQ score, RBS-r score and BMI, FSH, E2, F-G score, or acne score (**Table 2**).

**Table 1:** Comparisons of age, BMI, RBS-r score, total AQ score, and AQ subscale scores between the two groups.

	PCOS	Control	P value
n	100	100	
BMI	25.10 (4.15)^a^	23.03 (3.69)^b^	0.008**
age	30.50 (4.01)	32.47 (3.61)	0.000**
RBS-r score	15.01 (16.55)	11.34 (12.26)	0.071
total AQ score	61.16 (10.46)	55.57 (10.14)	0.000**
Social skills	11.60 (3.86)	10.18 (3.87)	0.010*
Attention switching	14.86 (3.30)	13.57 (3.28)	0.006**
Communication	9.61 (3.70)	7.79 (4.08)	0.001**
Attention to detail	13.85 (4.21)	13.11 (4.52)	0.232
Imagination	11.24 (3.65)	10.92 (3.35)	0.519

Data were reported as the mean (SD). *P value < 0.05; **P value < 0.01; ^a^Valid N=57; ^b^Valid N=50.

**Table 2:** Correlation between total AQ score, RBS-r score and sex hormone levels, F-G score, acne score, and BMI.

		Total AQ Score	RBS-r Score			Total AQ Score	RBS-r Score
BMI	r	0.08	0.12	LH	r	0.19	0.13
	P	0.397	0.228		P	0.010*	0.073
	n	107	107		n	195	195
FSH	r	-0.05	-0.09	E2	r	0.04	0.04
	P	0.454	0.237		P	0.627	0.603
	n	194	194		n	194	194
P	r	-0.03	-0.16	T	r	0.10	0.15
	P	0.738	0.046*		P	0.192	0.044*
	n	163	163		n	191	191
F-G score	r	-0.10	0.01	acne score	r	0.10	-0.15
	P	0.471	0.963		P	0.480	0.299
	n	53	53		n	53	53

*Statistical significance (P < 0.05).

**Figure 2:**
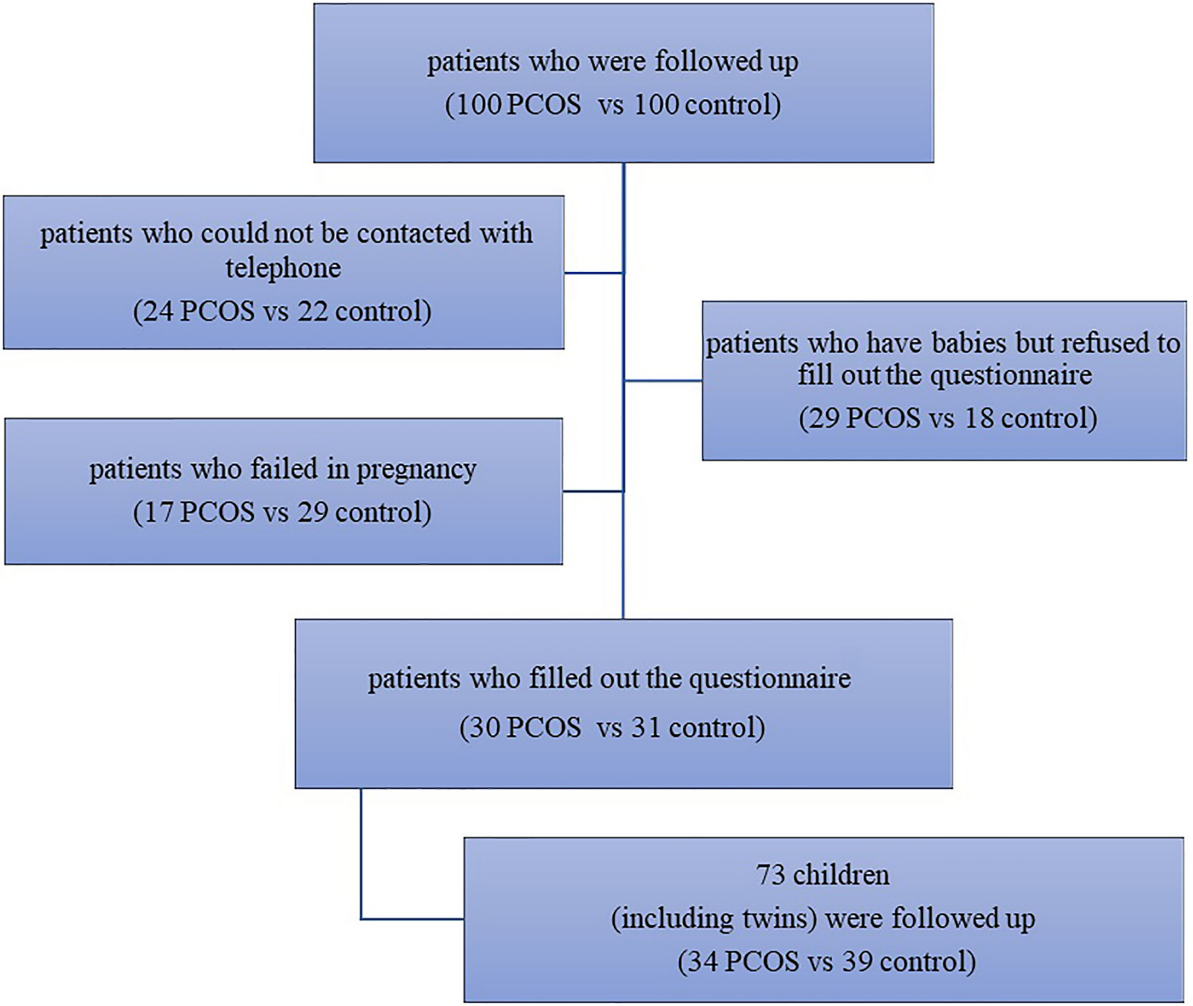
Flow chart of the follow-up study.

For the offspring follow-up study (**Figure 2**), 61 patients filled out the questionnaire (30 from the PCOS group vs 31 from the control group), 47 patients refused to fill out the questionnaire (PCOS vs control: 29% vs 18%, P=0.124), 46 patients failed in pregnancy (17 from the PCOS group vs 29 from the control group), and 46 patients could not be contacted with telephone number changes (24 from the PCOS group vs 22 from the control group). There was no significant difference between the two groups in delivery age, complications during pregnancy, special medication during pregnancy, negative events during pregnancy, delivery situation, or postpartum breastfeeding (**Table 3**). Due to the existence of twins, 73 children were followed up (34 from the PCOS group vs 39 from the control group). There was no significant difference in the age, AQ and ABC scores of children between the two groups (**Table 4**). Furthermore, after combining the two groups of data to analyse the correlation, it can be found that the AQ scores (P=0.036, r=0.248) and ABC scores (P< 0.001, r = 0.457) of offspring are positively correlated with the RBS-r scores of their mothers. Moreover, the ABC scores of offspring were positively correlated with the childbearing age of their mothers (P=0.008, r=0.306) (**Table 5**).

**Table 3:** Comparisons of childbearing age, pregnancy, delivery and breastfeeding conditions between the two groups of mothers at the follow-up.

	PCOS (n = 30)	Control (n = 31)	P value
**Age (years)**	32.41 (4.55)	33.66 (3.82)	0.252
**Complications during pregnancy**			
**Threatened abortion**	2 (6.67)	5 (16.13)	0.425
**Gestational diabetes mellitus**	8 (26.67)	8 (25.81)	0.939
**Gestational hypertension**	2 (6.67)	4 (12.90)	0.671
**Anaemia**	3 (10.00)	3 (9.68)	1.000
**Pollution of residence**	0 (0)	3 (9.68)	0.238
** Premature delivery**	2 (6.67)	4 (12.90)	0.671
** Infection**	1 (3.33)	0 (0)	0.492
**Medicine used during pregnancy**			
** Antibiotic**	1 (3.33)	3 (9.68)	0.612
** Progesterone**	14 (46.67)	18 (58.06)	0.373
** Multivitamin**	18 (60.00)	15 (48.39)	0.363
** Folacin**	25 (83.33)	26 (83.87)	1.000
** Others**	1 (3.33)	1 (3.23)	1.000
**Delivery condition**			
** Forceps delivery**	1 (3.33)	2 (6.45)	1.000
** Cesarean**	17 (56.67)	20 (64.52)	0.530
** Asphyxia neonatorum**	1 (3.33)	1 (3.23)	1.000
** Low-weight birth**	2 (6.67)	4 (12.90)	0.671
** High-weight birth**	0 (0)	2 (6.45)	0.492
**Negative events during pregnancy (e. g, death of relatives, divorce, etc.)**	0 (0)	1 (3.23)	1.000
**Breast-feeding**	27 (90.00)	30 (96.77)	0.354

Data were reported as the mean (SD), or n (%).

**Table 4:** Comparisons of age, ABC score, total AQ score, and AQ subscale scores between the two groups of children.

	PCOS (n = 34)	Control (n = 39)	P value
**Age (children)**	3.82 (1.25)	3.66 (0.59)	0.503
**ABC scores**	13.62 (22.96)	13.10 (13.20)	0.945
**total AQ scores**	58.68 (11.68)	60.18 (13.24)	0.905
**Social skills**	9.15 (5.49)	9.67 (4.12)	0.646
**Attention switching**	13.97 (3.64)	13.26 (3.09)	0.368
**Communication**	9.5 (4.30)	8.74 (4.78)	0.482
**Attention to detail**	15.41 (4.74)	17.51 (4.64)	0.061
**Imagination**	10.65 (4.21)	11.0 (3.15)	0.684

Data were reported as the mean (SD).

**Table 5:** Correlation between total AQ score of children, ABC score of the sex hormone levels of children and mothers, the child-bearing age of mothers, the total AQ score of mothers, and the RBS-r score of mothers.

		Total AQ Score of Children	ABC Score of Children			Total AQ Score of Children	ABC Score of Children
Total AQ score of mothers	r	0.134	0.010	RBS-r Score of mothers	r	0.248	0.457
	P	0.257	0.934		P	0.036*	0.000**
	n	73	73		n	73	73
FSH	r	-0.087	-0.173	LH	r	-0.187	-0.231
	P	0.470	0.147		P	0.115	0.051
	n	72	72		n	72	72
P	r	0.050	0.128	T	r	-0.093	-0.178
	P	0.695	0.316		P	0.468	0.163
	n	63	63		n	63	63
E2	r	0.166	0.064	Child-bearing age	r	0.022	0.306
	P	0.162	0.593		P	0.851	0.008**
	n	72	72		n	73	73

*P value < 0.05; **P value < 0.01.

## 4 Discussion

In this study, we found that women with PCOS had more autistic traits than those in the control group. The total AQ scores of women in social skills, attention switching and communication were significantly higher than those of the control group. The phenotypes were similar to the clinical manifestations of ASD, for example, social and language communication disorders. There was no difference between the two groups in attention to detail, imagination, or RBS-r scores. During the follow-up, PCOS patients were more likely to refuse the follow-up (29% in the PCOS group vs 18% in the control group). From another perspective, it showed that PCOS patients are more likely to have social dysfunctions.

In 2015, a population-based nationwide study in Sweden involving 23 748 ASD cases and 208 796 controls revealed that children of women with PCOS appear to have a higher risk of developing ASD (3). In 2018, another study based on Clinic Practice Research Datalink in the UK between 1990 and 2014 determined that women with PCOS and their children had a greater risk of autism (24). In addition, several studies showed that women with PCOS experienced higher rates of psychiatric diseases, such as depression and anxiety (25–29). It seems that there are some connections between ASD and PCOS. However, these two studies with large sample sizes were retrospective tracking trials on offspring without showing the sex hormone levels of mothers. In comparison, our research was a prospective study and tried to determine the associations between hormone levels and autistic traits in infertile patients with PCOS and their offspring. Our study provided more evidence that women with PCOS had more autistic traits, especially in social skills, communication, and attention switching. This suggested that women with PCOS are not only prone to have offspring with ASD but also suffer from social impairment themselves.

Why do PCOS patients have more autistic traits? PCOS patients are characterized by a high level of androgen, and ASD is currently considered to be associated with intrauterine hyperandrogen (30–32). Our study showed that the levels of LH and total testosterone in PCOS patients were significantly higher than those in the control group, which was consistent with known conclusions (1). Further correlation analysis revealed that autistic traits were associated with elevated total testosterone and LH.

Some studies have shown that testosterone easily binds to androgen receptors located in the cytoplasm through cell membranes and blood–brain barriers and then enters the nucleus, affecting the regulation of DNA transcription. It thus inhibits oxytocin (OXT), known as “social factors”, and oxytocin has been used to improve the clinical symptoms of ASD (33, 34). Testosterone can also affect the development of the nervous system and nerve contact by mediating programmed cell death, altering neurochemical mediators, and increasing the formation of dendritic synapses (35), and it might thereby affect social skills.

Regarding the relationship between LH and autistic traits, previous literature reported that the serum LH level in female patients with Asperger’s syndrome was higher than that in healthy women (36). LH stimulates ovarian follicular stromal cells to produce more androgen, which then affects the OXT system. This might be the mechanism by which LH affects social function.

In addition, we also found that progesterone was negatively correlated with the RBS-r score. In 2014, Whitaker-Azmitia et al. found that a decrease in progesterone in maternal serum was associated with an increase in the incidence of autism in offspring (37). He explained that progesterone could stimulate the production of more immune T cells, regulate autoimmune function, and combat neuroinflammatory effects. It thereby protects the brain development of offspring. Generally, progesterone rises after ovulation in women. The progesterone level in PCOS patients is low for a long time due to sparse ovulation. This may be one of the reasons why PCOS patients have more autistic traits.

AQ has been widely used in both clinical and research studies as a screen for diagnosis (38). In this study, the AQ scores, which represented sociability, did not show a significant difference between the two groups of children. This might be due to the sample size. However, our data showed that the social behaviors of children might be influenced by the rigid behaviors and delivery age of their mothers. Is age an independent high-risk factor affecting ASD in offspring? According to a literature review, compared with mothers aged 25 to 29, mothers aged ≥ 35 had an increased risk of autism in their offspring (RR=1.52), while mothers aged < 20 had a significantly reduced risk compared with mothers aged 25 to 29 (RR = 0.77). RR increases monotonically with increasing mother’s age. After considering the influence of father’s age and other potential confounding factors, this relationship still exists, which supports the independent relationship between mother’s older age and autism (39). According to another meta-analysis, when the age of mother and father increased by 10 years, the risk of autism of offspring increased by 18% and 21%, respectively (40). Our data showed that children’s ABC scores were positively correlated with their childbearing age (r=0.306, p=0.008), which was consistent with the existing literature results. At the same time, the children’s ABC score was positively correlated with the mother’s own rigid behavior (r=0.457, P < 0.001). This might be related to heredity, or it might be related to some behavioral influences of mothers in raising children. In addition, our study found that PCOS had a high rate of refusing follow-up (29% in the PCOS group vs 18% in the control group), which might be related to a low desire for social communication and the potential underestimation of offspring problems.

Finally, the limitations of our study must be considered: (1) PCOS is a heterogeneous disease. According to the Rotterdam criteria in 2003, the diagnostic evidence for PCOS is rare ovulation, hyperandrogenism, and polycystic ovarian changes. In other words, not all PCOS patients have hyperandrogenism, which leads to abnormal oxytocin and social behaviors. If it can be further subtyped, it will be more convincing. (2) People’s social behaviors are extremely complex. Whether social function can be fully evaluated by simply filling out the scale needs further confirmation. (3) This study mainly focused on infertile patients in the Reproductive Medicine Center, Peking University People’s Hospital. Infertility may be one factor causing social dysfunction. (4) Follow-up is in the form of an online questionnaire rather than an on-site examination of children, so the results may be biased.

## 5 Conclusion

In summary, our results demonstrated that PCOS patients have more autistic traits, which might be related to the elevation in testosterone concentration and luteinizing hormone levels, as well as the decline in progesterone levels. Moreover, the autistic traits in the offspring of PCOS patients might be related to the parental high delivery age and high tendency to autistic traits.

## Data Availability Statement

The original contributions presented in the study are included in the article/supplementary material. Further inquiries can be directed to the corresponding authors.

## Ethics Statement

The studies involving human participants were reviewed and approved by Peking University Institutional Review Board (Approval No. IRB00001052-15031). The patients/participants provided their written informed consent to participate in this study.

## Author Contributions

LJ is responsible for filling out questionnaires and writing articles. LT is responsible for recruiting patients. JY, XX and FQ are responsible for statistic data. RZ is responsible for reviewing data and revising articles. JW is responsible for revising articles. All authors read and approved the final manuscript.

## Conflict of Interest

The authors declare that the research was conducted in the absence of any commercial or financial relationships that could be construed as a potential conflict of interest.

## Publisher’s Note

All claims expressed in this article are solely those of the authors and do not necessarily represent those of their affiliated organizations, or those of the publisher, the editors and the reviewers. Any product that may be evaluated in this article, or claim that may be made by its manufacturer, is not guaranteed or endorsed by the publisher.
